# The neural representation of actions in Tourette syndrome as a window to decipher tics and their suppression

**DOI:** 10.1093/braincomms/fcad237

**Published:** 2023-09-01

**Authors:** Davide Martino, Christos Ganos

**Affiliations:** Department of Clinical Neurosciences, Cumming School of Medicine, University of Calgary, Calgary, AB T2N 4N1, Canada; Hotchkiss Brain Institute, University of Calgary, Calgary, AB T2N 4N1, Canada; Alberta Children’s Research Institute, University of Calgary, Calgary, AB T3B 6A8, Canada; Department of Neurology, Charité University Hospital, Berlin 10117, Germany

## Abstract

This scientific commentary refers to ‘Elevated representational similarity of voluntary action and inhibition in Tourette syndrome’, by Rae *et al*. (https://doi.org/10.1093/braincomms/fcad224).

**This scientific commentary refers to ‘Elevated representational similarity of voluntary action and inhibition in Tourette syndrome’, by Rae *et al*. (https://doi.org/10.1093/braincomms/fcad224).**

Investigating the mechanisms underlying tics and premonitory urges, the tightly connected core phenomena of tic disorders, inevitably leads neuroscientists to address several ‘old’ questions: (i) What makes actions voluntary and to what extent do they overlap with tics? (ii) What are the mechanisms underlying action generation and control and to what capacity do they contribute to tic generation? From a clinical standpoint, these questions are important for the fine-tuning of behavioural and neuromodulatory therapies to treat tics. This is especially relevant because the treatment response of tics can be variable across patients and often necessitates a combination of interventions.^1^

Historically, there have been many models to explain the occurrence of tics. Bliss’^2,3^ early behavioural model of tic control theorized that tics are voluntary motor responses to undesired somatosensory experiences known as premonitory urges, perpetuated because of negative reinforcement. Other theoretical frameworks conceptualized tics as prepotent actions that are not perceived as voluntary and are modulated by a voluntary inhibitory tic control that becomes increasingly efficient throughout development. For the generation of tics and premonitory urges, these later models called into play neural hubs that are primarily involved in the generation of voluntary actions and urges to act, i.e. medial frontal cortical areas—like the supplementary motor area (SMA) and the pre-SMA—the insular cortex and the striatum. Although experimental data have not yet provided a comprehensive mechanistic model of tic emergence, both clinical observations and behavioural data indicate that the interaction between the generation of actions, tics and volitional motor control lies at the core of tic pathophysiology.^3^

Voluntary tic inhibition may be mediated by a functional network that involves frontal cortical regions [inferior frontal gyrus (IFG) and pre-SMA] and the basal ganglia,^4,5^ thus suggesting potential overlap in neuroanatomical substrates between tic inhibition and action control. The right IFG and pre-SMA are engaged when preparing to stop an action and while stopping an action outright.^6^ Interestingly, this motor inhibition network is recruited across action-stopping tasks of variable complexity, suggesting that its involvement might be comparable during the suppression of both simple and complex tics.^7^ However, the neuroanatomical overlap between tic inhibition and action control poses an interesting neuroscientific question. How does the brain discern between voluntary actions and tics in order to achieve selective control of one type of motor output over the other?

To explore this discriminatory ability, Rae *et al*.^8^ chose an experimental paradigm that allowed them to compare action and inhibition processes between adults with Tourette syndrome and age-matched healthy volunteers independent of tics. Their study exploited the computational granularity of MRI-based representational similarity analysis (RSA), which discerns the similarity and dissimilarity of neural responses within a brain region based on the stimuli received or task conditions. They performed representational similarity analysis in relation to externally cued and internally chosen action and inhibition motor outcomes (right index button presses), in six pre-selected regions that, as summarized above, are putatively involved in tic-related mechanisms (right pre-SMA, right IFG, bilateral insular cortex, left caudate nucleus and left M1).

The study did not report any difference in behavioural performance on the externally cued selection of action and inhibition motor outcomes between the two groups of participants. Despite this equiperformance, the authors detected a greater representational similarity in Tourette syndrome subjects between externally cued action and inhibition, internally chosen action and inhibition, externally cued and internally chosen action and externally cued and chosen inhibition, all at the level of the right pre-SMA. Although statistical significance was reached only for the last of these contrasts, this finding suggests potentially similar neural activations between both action and inhibition and externally and internally cued motor programmes at the level of the pre-SMA. In turn, Rae *et al*.^8^ suggest that this indicates reduced accuracy in differentiating between these different action outcomes. Even with its limitations in statistical power, this study provides initial support to the authors’ hypothesis that the representation of action and inhibition within a network putatively controlling generation and inhibition of voluntary actions is more blurred in Tourette syndrome patients compared to healthy subjects.

At the same time, this observation stirs reflection and highlights several questions that are worthy of further investigation. The most obvious one, which the cross-sectional nature and the limited statistical power of this study do not allow to answer, is whether this blurring of contrast between motor outcomes is a primary, developmental ‘trait’ associated with Tourette syndrome or, rather, the ‘long-term outcome’ of exposure to tic behaviours. Interestingly, the representational similarity in pre-SMA did not correlate with tic severity or urge intensity, which were instead correlated with greater representational similarity in other regions (M1 and caudate, respectively), although only at a level of statistical trend.

Following up on the previous consideration, the design of this study does not allow to clarify whether a direct mechanistic link exists between the blurred neural representation of action and inhibition in pre-SMA and the voluntary control of tics. All the actions or action inhibitions processed by the participants of this experimental protocol were voluntary. By instructing Tourette syndrome participants not to intentionally suppress their tics, the voluntary motor outcomes used in the study were kept, to the best of the research team’s abilities, unconditioned by tic expression or tic suppression. However, it is possible that Tourette syndrome participants may have implicitly suppressed their tics during the scanning sessions, although this was not directly measured. Representational similarity of action and inhibition was not associated with tic expression during the experiment, indicating that the former is not a state marker of the propensity to tic. Furthermore, the presence of concurrent urges may have confounded the discrimination between externally and internally cued voluntary motor actions, but real-time urge intensity was not measured in the study. It is also striking that some of the pre-selected brain regions for this analysis, including pre-SMA, were not detected by recent lesion network maps identified from tic-inducing lesions.^9^ Finally, voluntary motor action/inhibition discrimination in this study was referred to a single body region (hand), therefore not exploring somatotopic differences that are important in tic disorders, given the inverse somatotopic gradient in tic expression and tic suppression.^10^

While the findings from Rae *et al*.^8^ may not directly expand our understanding of the mechanisms of tics and their suppression, they offer important insights with respect to the mechanisms of behavioural and neuromodulatory treatment strategies for tics. Both the habit reversal and the exposure–response prevention behavioural treatment approaches for tics instruct patients to treat internal cues (urges) as external commands to inhibit, ultimately teaching them to discern internal signals more accurately.^3^ Comparing representational similarity at the level of pre-SMA and of the IFG before and after these behavioural treatments might help elucidating the key mechanisms of behavioural therapy for tics or even assess whether this imaging marker is able to predict responsiveness to behavioural therapy. In a similar fashion, the change in representational similarity at the level of the pre-SMA in Tourette syndrome patients might support a rationale to use (facilitatory) non-invasive neuromodulation, for example targeting the IFG, to potentiate its inhibitory drive on motor output for treatment purposes. Ultimately, scoping the variability of prefronto–pre-SMA–basal ganglia connections across different subgroups and age groups of patients with tic disorders, and in relation to response to pharmacological therapies, will be necessary to confirm if the pattern of neural activation along this pathway is a useful physiomarker of tic disorders.
